# Attraction, Oviposition and Larval Survival of the Fungus Gnat, *Lycoriella ingenua*, on Fungal Species Isolated from Adults, Larvae, and Mushroom Compost

**DOI:** 10.1371/journal.pone.0167074

**Published:** 2016-12-09

**Authors:** Kevin R. Cloonan, Stefanos S. Andreadis, Haibin Chen, Nina E. Jenkins, Thomas C. Baker

**Affiliations:** 1 Department of Entomology, Penn State University, University Park, PA, United States of America; 2 Institute of Health and Environmental Ecology, Wenzhou Medical University, Wenzhou, Zhejiang Province, P. R. China; Chinese Academy of Agricultural Sciences Institute of Plant Protection, CHINA

## Abstract

We previously showed that the females of the mushroom sciarid, *Lycoriella ingenua* (Dufour, 1839) (Diptera: Sciaridae), one of the most severe pests of the cultivated white button mushroom, *Agaricus bisporus* (J.E. Lange) Emil J. Imbach (Agaricales: Agaricaceae), are attracted to the mushroom compost that mushrooms are grown on and not to the mushrooms themselves. We also showed that females are attracted to the parasitic green mold, *Trichoderma aggressivum*. In an attempt to identify what is in the mushroom compost that attracts female *L*. *ingenua*, we isolated several species of fungi from adult males and females, third instar larvae, and mushroom compost itself. We then analyzed the attraction of females to these substrates using a static-flow two choice olfactometer, as well as their oviposition tendencies in another type of assay under choice and no-choice conditions. We also assessed the survival of larvae to adulthood when first instar larvae were placed on each of the isolated fungal species. We found that female flies were attracted most to the mycoparasitic green mold, *T*. *aggressivum*, to *Penicilium citrinum* isolated from adult female bodies, and to *Scatylidium thermophilium* isolated from the mushroom compost. Gravid female flies laid the most eggs on *T*. *aggressivum*, *Aspergillus flavus* isolated from third instar larval frass, *Aspergillus fumigatus* isolated from adult male bodies, and on *P*. *citrinum*. This egg-laying trend remained consistent under no-choice conditions as females aged. First instar larvae developed to adulthood only on *S*. *thermophilium* and *Chaetomium* sp. isolated from mushroom compost, and on *P*. *citrinum*. Our results indicate that the volatiles from a suite of different fungal species act in tandem in the natural setting of mushroom compost, with some first attracting gravid female flies and then others causing them to oviposit. The ecological context of these findings is important for creating an optimal strategy for using possible semiochemicals isolated from these fungal species to better monitor and control this pestiferous mushroom fly species.

## Introduction

The fungus gnat, *Lycoriella ingenua* (Dufour 1839) (Diptera: Sciaridae), formerly known as *Lycoriella mali* [1], causes some of the most severe insect damage to cultivated white button mushrooms, *Agaricus bisporus* (J.E.Lange) Emil J. Imbach (Agaricales: Agaricaceae), in the United States. White button mushrooms are a high value crop, with an estimated 394,650 tons produced between 2012 and 2013 grossing nearly US $1.4 billion [2]. Fungus gnats are especially effective pests of white button mushrooms considering this pests’ low economic threshold [3, 4], high fecundity [5], and the range of damage characteristics it inflicts on the crop. This damage includes: direct larval feeding on developing *A*. *bisporus* mycelia in the growing compost media [6–8]; larval competition with developing *A*. *bisporus* mycelia for nutrients in the compost [9]; and a reduction in mycelial growth due to larval excrement (frass) [10]. Mushroom growers have reported that *L*. *ingenua* adults vector into mushroom houses the green mold, *Trichoderma aggressivum*, one of the most severe fungal pathogens of *A*. *bisporus*, [11]. Although no experiments have examined the ability of *L*. *ingenua* to vector *T*. *aggressivum*, scanning electron microcscopy images revealed that adult *L*. *ingenua* adults carry spores of the plant pathogen *Fusarium fungicola* on their bodies [12] thus may potentially vector the spores of other pathogenic fungi.

Methods to control this fly include compost drenches with the insecticide imadocloprid [13], the juvenile hormone (JH) analog methoprene, and the chitin synthase inhibitor diflubenzuron [14]. However, several factors have shown the necessity for changing standard pest management practices for this species. Early reports of rapid resistance development by the flies to pyrethroid insecticides [15–17], the difficulty in excluding flies from mushroom houses, and a growing consumer demand for chemical-free mushrooms [18], have all contributed to a realization that novel, sustainable pest management tools are necessary to provide mushroom growers with biorational ways to keep pest populations down.

For instance, recent work has resulted in the successful isolation and partial identification of a female-produced sex pheromone component of *L*. *ingenua* that is attractive to male flies [19]. Although this component is clearly different from the pheromone that was previously misidentified [20] and later shown to be behaviorally inactive [21] the precise stereoisomeric structure of this compound needs to be characterized before it can be synthesized and used for monitoring and possibly mass trapping of males within and outside of mushroom houses. What would further add to an arsenal of semiochemical pest management tools for *L*. *ingenua* would be if attractants for female flies could be found for use in monitoring and mass trapping of females. Thus, the goal of this research was to isolate and identify potential fungal sources from the mushroom growing compost that are best at attracting gravid female *L*. *ingenua* flies. We wanted to make sure that in our efforts we would specifically include fungal species that are suspected of being vectored in and out of the mushroom-growing compost by both adult and larval flies.

We previously showed, using a static flow olfactometer, that gravid female *L*. *ingenua* flies were highly attracted to *T*. *aggressivum* both in culture and growing on spawned compost [22]. “Spawned compost” refers to mushroom compost that has *A*. *bisporus* mycelia already developing in it. These results suggest that the belief by growers about *L*. *ingenua* vectoring green mold spores into a growing house may be true, and the flies may be responsible for high infestations of *T*. *aggressivum*. We also showed that gravid female *L*. *ingenua* flies are equally attracted to spawned compost and unspawned compost (compost with no *A*. *bisporus* mycelia). Attraction to unspawned compost was significantly reduced when it was sterilized, suggesting that gravid females are attracted to actively metabolizing microorganisms within the unspawned compost, not the developing *A*. *bisporus* mycelia. These results support early work showing that *L*. *ingenua* development on sterilized compost is significantly reduced versus non-sterilized compost [23]. Because of these findings, Kielbasa and Snetsinger (1981) suggested that, “*L*. *mali* (now *L*. *ingenua*) obtains dietary supplementation from bacteria and/or fungi which survive pasteurization.” Considering that the microbial community of mushroom compost is both diverse and temporally dynamic [24] it is reasonable to assume that females are attracted to the volatile profile of one or more of these metabolizing microorganisms.

As a result of the abovementioned findings we hypothesized that female flies might be attracted to one or several of the actively metabolizing fungal species we had found to be present in mushroom compost. Previous work showed that gravid *L*. *ingenua* females were differentially attracted to fungal species grown on a variety of substrates [25]. The authors suggested that this differential attraction was due to the different suite of volatiles produced as a result of the same fungal species utilizing different substrates. We therefore designed experiments to use some fungal cultures that we isolated from compost that we now grew only on potato dextrose agar (PDA) to see the extent to which female *L*. *ingenua* would be attracted to them and lay eggs in them. We also included fungal species that we were able to obtain from the bodies of males and females that had been collected from compost, given previous evidence that *L*. *ingenua* carry other fungi on their bodies [12]. Such phoretic fungi might have extra importance in the lives of *L*. *ingenua* and might therefore be more attractive to females or provide more powerful oviposition cues. For these experiments we used two-choice attraction assays, two-choice oviposition assays, and no-choice oviposition assays in order to try to find fungal species that might be more attractive to female *L*. *ingenua* than *T*. *aggressivum* and might emit volatiles that can be isolated and identified for possible use in monitoring traps in mushroom houses.

## Materials and Methods

### Insects

The fungus gnats used in this study were from a three-year-old laboratory colony maintained at the University Park Campus of The Pennsylvania State University, Department of Entomology, and were positively identified by Dr. Seunggwan Shin (North Carolina State University, Department of Entomology, Raleigh, NC). All *L*. *ingenua* culturing methods including colony initiation were identical to those described by Cloonan et al., (2016). Flies were reared on a mixture of unspawned mushroom compost and nitrogen supplement (100:1) in an environmental growth chamber at 21°C, 70% r.h., and a L12:D12 photoperiod. Nine plastic Solo cups (355-ml, Solo, MI, USA) were filled to the top with the unspawned compost–nitrogen supplement mixture and placed into a mesh cage (BioQuip, CA, USA; 30 x 30 x 30 cm) with approximately 100 male and 100 female *L*. *ingenua* flies. These cups were then left under colony temperature and relative humidity conditions for two days to allow the flies to mate and the females to oviposit in the compost mixture provided. After two days, the cages were covered with plastic autoclave bags to prevent the compost from drying out. The cages were left under colony conditions until future adult flies emerged approximately 21 days later.

To obtain females of a specific age for experimentation, newly emerged adults (0–12 hours old) present on the screened walls of their cages had their nine plastic Solo emergence cups removed, and then a paper towel with a 10% table sugar solution was provided at the bottom of the cage to allow adults to feed *ad libitum*. The cages were then kept under colony conditions for the appropriate amount of time in order to obtain cages of gravid females of a specific age for different experiments. We found that adults would live under these conditions up to 12 days with ca. 50% of adults dying by the eighth day (unpublished data).

### Fungal Cultures

#### Fungal species present in mushroom compost

Two cultures, *Scytalidium thermophilum* and *Chaetomium* sp., were obtained from the Plant Pathology Department at Penn State University. These two fungal species were chosen because of their relatively high abundance in mushroom compost [26] and that therefore they might be likely candidates for attracting female *L*. *ingenua* to the compost. A culture of *T*. *aggressivum* was also obtained from the Plant Pathology Department at Penn State University and was used as a control in all two-choice attraction and oviposition experiments because it is the most attractive substrate to gravid females that we have found thus far. Our goal was to isolate fungal species that were highly attractive to *L*. *ingenua* females, and to find those that might be even more attractive than *T*. *aggressivum*.

#### Fungal species isolated from adult flies

All fungi obtained from adult flies for these experiments were cultured on plates of potato dextrose agar (PDA) (8.5 cm in diameter; Crystalgen Inc, NY, USA) that were kept in an environmental growth chamber at 21°C, 70% r.h., and a L12:D12 photoperiod for 1 hr. In order to ensure that the fungal species we isolated from flies actually did originate from the bodies of adult flies and not from contamination from elsewhere in the environment, we performed three different culturing treatments: 1) placing surface-sterilized adult flies on PDA; 2) placing non-sterilized adult adult flies on PDA; and 3) exposing empty plates of PDA to our work station environment. To obtain surface-sterilized flies, ten male and ten female newly emerged adults from the mesh colony cages were aspirated and immediately placed in a freezer (18°C) for 1 min to temporarily immobilize them. They were then set on top of two layers of dry paper towels and drenched in a 5% bleach solution, then immediately rinsed with deionized (DI) water. The adults were then transferred to another set of paper towels to dry. These dry, surface-sterilized flies were then individually placed on plates of PDA (8.5 cm in diameter) and kept in an environmental growth chamber at 21°C, 70% r.h., and a L12:D12 photoperiod for 1 hr. After 1 hr, sterilized flies were discarded and the plate was covered with parafilm, then placed back into the growth chamber for 72 hr to monitor for colony formations.

To obtain non-surface-sterilized adults, ten male and ten female flies were treated the same way except they were not drenched in bleach or DI water before being placed on plates of PDA. They were then put into the growth chamber under the same conditions. We also exposed 10 plates of PDA to the open air of our work station for ca. 10 sec, as well as exposing them briefly to the clean tips of the brushes used to transfer sterilized and non-sterilized adults to ensure any fungal growth we observed originated from the adult fly bodies. After 72 hr Individual fungal colonies from all treated plates were then isolated under sterile conditions by placing them onto new Petri plates of PDA that were kept under colony conditions for ca. 2 weeks or until we could verify visually that we had isolated a single individual fungal species.

#### Fungal species isolated from larvae

To obtain sterilized larvae, we collected ten third instars reared on unspawned compost and drenched them in bleach and DI water the same way as we treated the sterilized adults above, except larvae were not cold-treated first to immobilize them as we had done for adults. Larvae were then transferred individually to plates of PDA that were then placed in an environmental growth chamber at 21°C, 70% r.h., and a L12:D12 photoperiod for 1 hr. After 1 hr, sterilized larvae were removed from the plates of PDA which were then covered with parafilm and placed back into the growth chamber for 72 hr to monitor for colony formations.

Ten non-sterilized third instars were treated the same way as the larvae above except they were not drenched in bleach or DI water before being placed on plates of PDA and put into the growth chamber under the same conditions. After 72 hr individual fungal colonies from all treated plates were then isolated under sterile conditions by placing them onto new Petri plates of PDA and kept under colony conditions for ca. 2 weeks, or until we could verify visually that we had isolated a single individual fungal species.

#### DNA Extraction, PCR, Sequencing and Identification of Isolated Fungi

DNA was extracted from mycelial mats grown in potato dextrose liquid media. Small plugs (2 mm in diameter) of each culture, grown on plates of PDA for 14 days, were placed into 250 ml flasks of potato dextrose liquid media and allowed to grow at 27 ± 1°C on a shaker rotating at 180 rpms for 14 days. After 14 days mycelial mats were harvested by filtering the liquid media through filter paper (Whatman® qualitative filter paper, Grade 1, 42.5 mm diam) using gravity and stored at -80°C. Samples were then freeze-dried in a Virtis Advantage XL Lyophilizer, held for 5 hr after the pressure dropped below 200 mT, and slowly increased to room temperature over 7 hours. DNA was extracted from each sample by grinding approximately 40 mg of tissue in liquid nitrogen then adding 600 μl of Nuclei Lysis Solution (1% SDS, 10 μl EDTA). These samples were then incubated at 65°C for 30 min. After incubation, 1 μl of 1 mg/mL RNase was added and samples were then incubated at 37°C for 15 min. Samples were then cooled to room temperature for 5 min. To this cooled sample 200 μl of a 10M ammonium acetate solution was added. The sample was then cooled and incubated for 5 min on ice, then centrifuged at 16,000 x g for 3 min. The supernatant was then transferred to clean Eppendorf tubes containing 600 μl of room temperature isopropanol and centrifuged at 16,000 x g for 3 min. A 70% ethanol solution was then added to the samples and they were centrifuged at 16,000 x g for 1 min. The pellet was then air dried and suspended in 100 μl of sterile water and rehydrated overnight at 4°C.

These samples and two sets of primers (Bt2a/Bt2a) and (cmd5/cmd6) were amplified by PCR. These PCR products were then sequenced by Sanger DNA sequencing (Applied Biosystems 3730XL) at Genomics Core Facility service at The Pennsylvania State University. Results were edited using Geneious 9.0.5 software. These were then compared to Genbank to determine their identity.

### Two-Choice, Static Flow Olfactometer Attraction Assays

**Fig 1:**
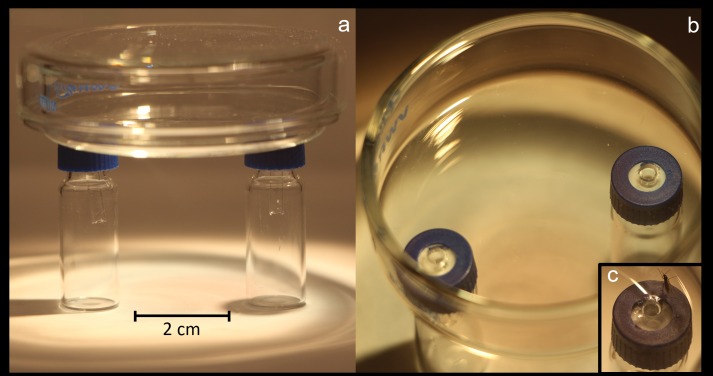
Two-choice olfactometer apparatus. (a) The olfactometer consists of a 5-cm-diameter glass Petri dish release arena attached to two 2-ml glass vial pitfall traps spaced 3.5-cm apart through two 3-mm-diameter holes. A 4-mm-diameter, 1.5-cm-long glass tube extends into each of the two 2-ml glass vial pitfall trap lids. These glass tubes are affixed into the lids of the 2-ml glass vial pitfall traps. (b) The tips of the tubes extend out of the 2-ml glass vial pitfall traps and are positioned directly under 3-mm diameter holes drilled into the release arena floor. Because the tubes are 1-mm diameter larger than the drilled holes, the release arena is able to be set directly over these holes such that they lay flush with the release arena floor. This is critical in facilitating female *Lycoriella ingenua* entrance into the pitfall traps. (c) A single female fly is shown here resting next to the glass tube leading to a 2-ml glass vial pitfall trap.

To examine the relative attractiveness to gravid females of all isolated fungal species, we used a modified static-flow two choice olfactometer previously described in Cloonan et al. (2016) (Fig 1). Each olfactometer was precisely machined to be as identical as possible. Each one consisted of a glass Petri plate “release arena” (VWR, Radnor, PA, USA), 5 cm in diameter, connected at the bottom to two 2 ml glass vials (12 x 32 mm) (Supelco, Bellefonte, PA, USA) acting as pitfall traps. The pitfall traps were connected to the release arena by two 3 mm diam. holes spaced 3.5 cm apart that had been drilled into the release arena floor. The pitfall traps were then connected to these holes in the arena by means of 4 mm i.d. glass tubes (100–200 μl microdispensor replacement tubes; Drummond Scientific, Broomhall, PA, USA). The tubes were 3 cm in length and open at both ends. These tubes were affixed into the lid (screw thread PTFE/Silicone septum 9 mm in diameter) of the pitfall traps such that they extended 1 cm into the interior of the traps. Because the outer diameter of each if these glass connecting tubes was 1 mm wider than the 3 mm holes drilled into the release arena floors, the release arenas rested directly on top of the two pitfall traps, with an opening that went directly from the release arena floor into the pitfall traps (Fig 1B). The utility of these static-flow olfactometers lies in the fact that once a gravid female fly makes her choice and enters one of the pitfall traps containing an attractive substrate, she is unable to pass back through to the release arena and her choice can be easily and accurately recorded.

Each pitfall trap contained one disk of test substrate punched out of the above-described 2-week-old fungal cultures grown on PDA. These disks (7 mm in diameter) were punched out of PDA cultures using the wide end of a yellow 1–200 μl pipette tip (VWR International, Radnor, PA, USA). For each two-choice test, one pitfall trap always contained *T*. *aggressivum* and the other pitfall trap contained one of the other five fungal cultures described. Individual 2-day-old gravid female flies were aspirated into the release arena, whose holes were then gently positioned so that they rested over the two connecting tubes of the two pitfall traps that contained disks of fungal cultures. Once set in position, the olfactometers were placed under colony conditions and females were allowed to make their choice over a 12 hr period of 6 hr of light and 6 hr of dark. At the end of each 12 hr experiment the locations of females were recorded and the flies were then discarded. Fungal disks were discarded, and the release arenas and pitfall traps were then soaked and washed in detergent and water, rinsed, and then soaked in a 70% ethanol bath for 1 hr before being air dried overnight before re-use.

These olfactometer experiments were conducted in 30 concurrently-running olfactometers each night. Paired fungal cultures were presented to individual female flies in a complete-block replicate having the six following treatments:

1. *Trichoderma aggressivum* versus *Aspergillus niger* isolated from adult male flies
2. *Trichoderma aggressivum* versus *Aspergillus flavus* isolated from larval frass
3. *Trichoderma aggressivum* versus *Aspergillus fumigatus* isolated from adult female flies
4. *Trichoderma aggressivum* versus *Penicillium citrinum* isolated from adult female flies
5. *Trichoderma aggressivum* versus *Scatylidium thermophilum* obtained from Plant Pathology at The Pennsylvania State University
6. *Trichoderma aggressivum* versus *Chaetomium* sp. obtained from Plant Pathology at The Pennsylvania State University

Five replicates were run each night (1 female per each of 6 treatments X 5 replicates = 30 females per night) over 12 nights, using a different batch of females each night. Thus, overall, 60 different females were tested for their response to each treatment (the six fungal culture choices) over 12 nights.

### Two-Choice Oviposition Assays

We also examined the oviposition preferences of individual two-day-old gravid females in response to the above-described treatments, i.e., the six fungal culture pairings, grown on PDA. These assays were performed in covered 5-cm-diameter Petri dishes from the same manufacturer as used in the olfactometer experiments above. These Petri dishes had no holes drilled into their floors. For each replicate two 7-mm-diameter disks of two-week-old fungal isolates grown on PDA, obtained as described above, were placed 4 cm apart on the Petri dish floor. An individual two-day-old gravid female was then aspirated into the Petri dish that was then promptly covered with its glass lid and placed under colony conditions for 6 hr of light and 6 hr of dark. At the end of each 12 hr experiment, the numbers of eggs were counted on each fungal disk, and the disks were then discarded. The Petri dishes were then soaked and washed with detergent-water, rinsed, then soaked in a 70% ethanol bath for 1 hr before being air dried overnight before re-use.

Fungal cultures were presented to 18 individual female flies in the described paired combinations each night, forming 3 complete-block replicates of the six treatments each night. The experiment was run over 5 nights, using a different batch of females each night. Thus, overall, 15 different females were tested for their response to each treatment over 5 nights.

### No-Choice Assays

We also performed no-choice oviposition assays to determine the degree to which females, if they had no choice, would oviposit on both the preferred and non-preferred fungal isolates that were used in the two-choice oviposition assays above. We conducted these experiments because the attractive presence of *T*. *aggressivum* in every two-choice oviposition assay above may have affected our ability to measure females’ acceptance or rejection behaviors related to these fungal substrates. In these no-choice assays we recorded the daily number of eggs laid by females to see if their egg deposition might become greater even on less-preferred fungi as females matured and died. These assays were performed in the same glass Petri dishes that were used in the two-choice oviposition assays. Individual two-week old disks (7 mm in diameter) of each of the following fungal cultures were placed directly in the center of the glass Petri plate:

1. Blank PDA
2. *Aspergillus fumigatus* isolated from adult female flies
3. *Aspergrillus flavus* isolated from larval frass
4. *Trichoderma* *aggressivum*
5. *Chaetomium* sp. obtained from Plant Pathology at The Pennsylvania State University
6. *Penicillium citrinum* isolated from adult female flies
7. *Scatylidium thermophilum* obtained from Plant Pathology at The Pennsylvania State University
8. *Aspergillus niger* isolated from adult male flies

The goal of these no-choice experiments was to examine whether female flies would oviposit on less-preferred fungal isolates as they aged. However, we were unable to monitor the oviposition behavior of individual female flies throughout their lifetime, because even unmated flies will oviposit all of their eggs on a preferred substrate and die within a few hours (unpublished data). Because of this we developed a method to monitor the oviposition behavior of replicated individual flies of the same age under no-choice conditions, from newly emerged females to seven-day-old females. To obtain mated females of the same age, individual cages of male and female flies of a specific age were held under colony conditions until they were used for experimentation. All of the females in each one of these cages were thus considered mated as they were held at a ratio of ca. 50:50 male to females within each cage of specifically aged individuals. Individual female flies, between the ages of newly emerged to seven-days-old, were aspirated into a Petri dish containing a single fungal culture disk placed in the center. The Petri dishes were then placed under colony conditions for 12 hr (6 hr of light and 6 hr of dark). At the end of each 12 hr experiment the numbers of eggs were counted on each fungal disk, and the disks were then discarded. The release arenas were then cleaned as before.

Three replicates with respect to female age were run during each no-choice oviposition assay (one female from each of the seven age classes X 3 replicates = 21 females per night), using a different fresh batch of females aged 1–7 days each session. Thus we used 21 Petri-dish assay chambers during each 12-hr session. Individual females from all 7 age groups were tested during each session against three of the eight different fungal cultures in our regime (including blank PDA). Due to space and supply constraints we could not test all the fungal cultures simultaneously against all seven age-group females during a single 12-hr session. The array of three fungal treatments used in each session was chosen randomly until 15 females had been assayed against each culture during five different sessions.

### Larvae-to-Adult Survival Assays

We transferred individual first instar larvae to agar disks of each isolate to examine larval survival to adulthood. First instar larvae were carefully transferred to two-week-old cultures grown on PDA (7 mm in diameter) that were placed in a Petri dish (the same as was used in the no-choice oviposition assays; 5 cm in diameter). The Petri dishes were then covered in parafilm and placed back in colony conditions at 21°C and a 12:12 photoperiod (light:dark). These dishes were then monitored each day for larval development and subsequent adult emergence. 30 individual first-instar larvae were tested per isolated fungal species and a PDA control. For each replicate, five first-instars were placed on each of the seven fungal cultivars plus blank PDA and observed through to adulthood or death. Six of these replicates were run over the course of the experiment.

### Statistical Analysis

All statistics were conducted using Prism 5.0 software (GraphPad Software Inc., San Diego, CA, USA). All datasets were first analyzed for normality via the D'Agostino & Pearson Omnibus Normality Test. Differences among proportions of female flies found in individual pitfall traps for the two-choice olfactometer experiments were assessed via Chi-square goodness of fit test after correcting for continuity with Yates’ correction factor. Non-responders remaining in the release chamber were excluded from these analyses. The two-choice oviposition data were compared via the Mann–Whitney *U* test. The differences in mean egg numbers among each age group for the no-choice oviposition data were analyzed via the Kruskal-Wallis test, and differences in means were then compared via Dunn’s Multiple Comparisons. The differences in mean survival from larvae to adult were analyzed via the Kruskal-Wallis test, and differences in mean survival on different fungal substrates were then compared via Dunn’s Multiple Comparisons.

## Results

Sequence comparisons referencing GenBank positively identified the following fungal isolates to species: *Aspergillus flavus* was isolated from larval frass, *A*. *niger* from adult male bodies, and *A*. *fumigatus* from adult female bodies, and *Penicillium citrinum* was isolated from adult female bodies.

**Fig 2:**
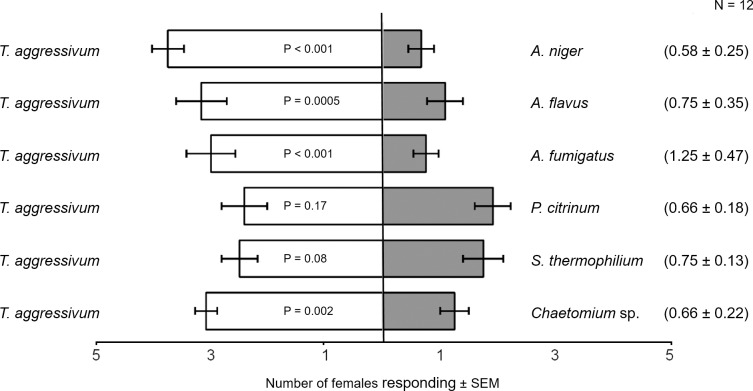
Two-choice attraction assays. Mean number (± SEM) of two-day-old gravid female *Lycoriella ingenua* flies attracted to various pure fungal cultures grown on potato dextrose agar in two-choice, static-flow olfactometer assays. Each horizontal bar is the mean of responses of 5 females to each treatment over 12 replicates (N = 12). The mean number of non-responders (± SEM) for each combination is included in parentheses to the right. Female choices for each pair of cultures were analyzed via chi square. All non-responders were excluded from the analysis.

**Fig 3:**
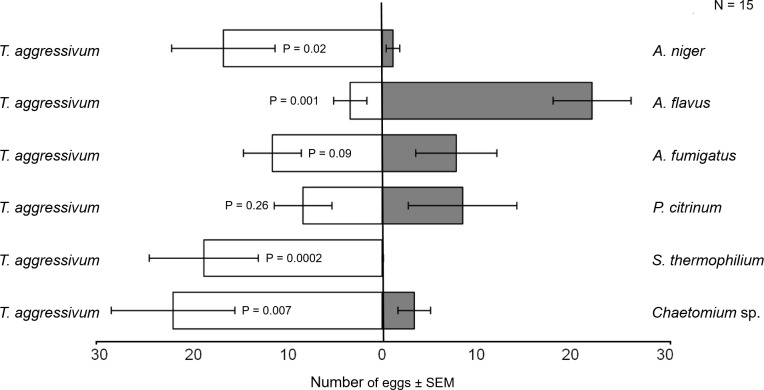
Two-choice oviposition assays. Mean number (± SEM) of eggs laid by two-day old gravid female *L*. *ingenua* flies on various pure fungal cultures grown on potato dextrose agar in two-choice oviposition assays. Each horizontal bar shows the mean number of eggs laid on the two choices of fungi by 15 two-day-old gravid female flies, tested in groups of three in three individual chambers with 5 different cohorts of females being tested over 5 different nights. All data were non-normally distributed and differences between the mean number of eggs deposited on each pair of fungal cultures were analyzed via the Mann–Whitney *U* test (two-tailed, df = 14).

Females were attracted more to *T*. *aggressivum* than to *Aspergillus niger* in two-choice pitfall traps (P < 0.0001; χ^2^ = 22.41) (Fig 2). This greater relative attraction to *T*. *aggressivum* was reflected also in our two-choice oviposition assays, in which female flies laid significantly more eggs on *T*. *aggressivum* than on *A*. *niger* (P = 0.02) (Fig 3). A different relationship between attraction and oviposition was seen when females had to choose between *T*. *aggressivum* and *A*. *flavus* isolated from third instar frass. In this case, females were more attracted to *T*. *aggressivum* than to *A*. *flavus* in two-choice olfactometer assays (P = 0.0005; χ^2^ = 10.95) (Fig 2), but they oviposited significantly more eggs on *A*. *flavus* than on *T*. *aggressivum* (P = 0.001) (Fig 3).

For the third fungal species isolated from *L*. *ingenua*, females were more attracted to *T*. *aggressivum* than to *A*. *fumigatus* isolated from adult female bodies (P < 0.001;; χ^2^ = 15.02) (Fig 2), but they oviposited similar numbers of eggs on these two fungal species (P = 0.09) (Fig 3). In response to *P*. *citrinum*, the *Penicillium* fungal species isolated from adult female flies, females were equally attracted to *P*. *citrinum* and to *T*. *aggressivum* (P = 0.17; χ^2^ = 1.42) (Fig 2), and they also laid similar numbers of eggs on both of these fungal species in the two-choice oviposition assay (P = 0.26) (Fig 3).

For one of the fungal species isolated from compost, *S*. *thermophilium*, females were equally attracted to *T*. *aggressivum* and *S*. *thermophilium* in our two-choice olfactometer tests (P = 0.08; χ^2^ = 2.42) (Fig 2). *Scatylidium thermophilium* is the fungal species known to be present in high abundance in mushroom compost and that we did not find on adult bodies. Despite these equal attraction levels, females laid significantly more eggs on *T*. *aggressivum* (P = 0.0002) (Fig 3) than on *S*. *thermophilium*, almost completely avoiding oviposition on this species in two-choice oviposition assays. In response to the *Chaetomium* sp., another fungus we isolated from mushroom compost but that we could not isolate from adults’ bodies, females were significantly more attracted to *T*. *aggressivum* than to *Chaetomium sp*., in the olfactometer assays (P = 0.002; χ^2^ = 8.42) (Fig 2). The attraction results were reflected later in the two-choice oviposition assays: when given a choice between these two species, females laid significantly more eggs in *T*. *aggressivum* than *Chaetomium* sp. (P = 0.007) (Fig 3).

**Fig 4:**
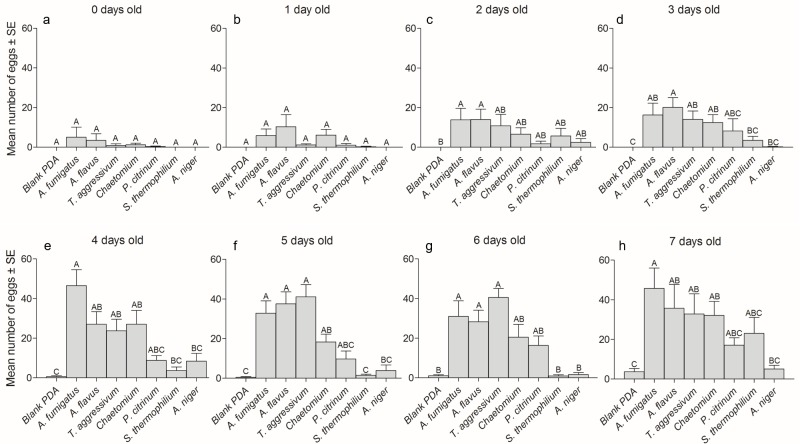
No-choice oviposition assays. Mean number (± SEM) of eggs laid by 0- (day of emergence) to-7 day-old female *L*. *ingenua* flies on various pure fungal cultures grown on potato dextrose agar in no-choice oviposition assays. A total of 15 flies of a particular age were tested for their tendency to lay eggs on each of the 8 fungal cultures under no-choice conditions, resulting in a total of 120 females (15 flies x 8 fungal cultures) of each age that were tested for oviposition on all fungal cultures. There were 8 age groups tested (panels a-h), and thus 840 different female flies were used in this experiment. All data were non-normally distributed and differences among mean egg numbers within each age group were analyzed via the Kruskal-Wallis test. Differences between mean egg numbers for each age group were compared using the Dunn’s Multiple Comparisons Test. No comparisons were made between mean numbers of eggs in different age groups. Different letters above histograms within the same age group indicate a significant difference (df = 14; P < 0.05).

The no-choice oviposition experiment showed females’ pure responses to invest or not invest significant numbers of eggs on various fungal substrates. Female *L*. *ingenua* that were newly emerged to three-days-old laid similar, small numbers of eggs on all fungal cultures under no-choice conditions (P > 0.5) (Fig 4A–4D). However, after reaching the age of four-days-old, females began laying significantly more eggs on those fungal substrates they had shown a preference for in the two-choice oviposition assays, which included *A*. *fumigatus*, *A*. *flavus*, *T*. *aggressivum*, *Chaetomium* sp., and *P*. *citrinum*. The non-preferred fungi from those assays, *S*. *thermophilium* and *A*. *niger* (P < 0.5) (Fig 4E–4H), evoked very little oviposition even from older females in these no-choice tests.

Thus, in general, those fungal species that were not preferred for oviposition under two-choice conditions had very few eggs laid on them under no-choice conditions by females of all age groups. For example, females laid almost no eggs on *A*. *niger* under no-choice conditions across all age groups and they had almost completely avoided laying eggs on *A*. *niger* in the two-choice oviposition assays. There were no statistically significant differences in egg numbers between blank PDA and *A*. *niger* across all age groups (Fig 4A–4H). Even at the end of their lives, at seven-days-old (Fig 4H), females chose to lay their eggs on the plastic sides of Petri dishes before they died, rather than laying them on *A*. *niger*. A similar result was seen in no-choice oviposition tests for *S*. *thermophilium*. There were no significant differences in the very low egg numbers deposited by females aged 1–7 days on *S*. *thermophilium* compared to either blank PDA or *A*. *niger* (Fig 4H).

There were two curious anomalies in no-choice oviposition results compared to the two-choice assays. In the two-choice assays, both *T*. *aggressivum* and *P*. *citrinum* had received similarly large numbers of eggs. Under no-choice conditions *P*. *citrinum* received numbers of eggs similar to the blank PDA controls and to *T*. *aggressivum* across all age groups (Fig 4A–4H). However, *T*. *aggressivum* only received similar numbers of eggs to the blank PDA control from newly emerged, one-day-old, and two-day-old females (Fig 4A–4C). Similar results were seen for *Chaetomium* sp., the species of fungus found in high abundance in mushroom compost. Although not preferred for oviposition compared to *T*. *aggressivum* in two-choice assays, it was readily accepted as an oviposition site by females under no choice conditions. Our blank PDA controls received very few eggs from female flies across all age groups.

**Fig 5:**
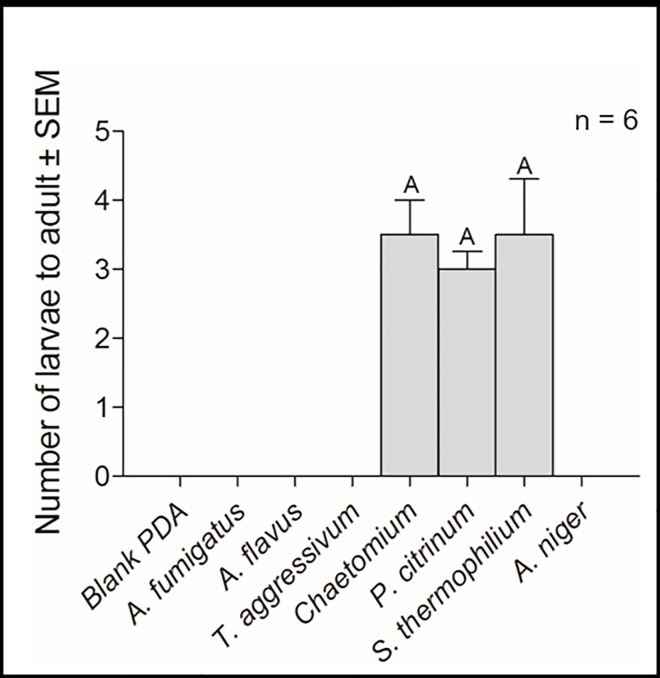
Larval survival assays. Mean survival (± SEM) of *Lycoriella ingenua* larvae to adults on various pure fungal cultures grown on potato dextrose agar. Each histogram is the mean survival of 5 newly emerged first instar larvae, replicated from 6 different cohorts of larvae. All data were non-normally distributed and differences among mean survival on different fungal cultures were first analyzed via the Kruskal-Wallis test. Differences between survival on different fungal cultures were compared with the Dunn’s Multiple Comparisons Test. Different letters above bars indicate a significant difference (df = 5; P < 0.05).

No larvae survived past the first instar on pure cultures of *A*. *flavus*, *A*. *niger*, *A*. *fumigatus*, or *T*. *aggressivum* (Fig 5). On *P*. *citrinum* 60% of the first instars survived to adulthood, 73% survived to adulthood on *Chaetomium* sp., and 70% survived to adulthood on *S*. *thermophilum* (Fig 5). No larvae survived past the first instar on our PDA controls.

## Discussion

There is a growing body of experimental evidence showing that many species of insects are attracted to microbial volatile organic compounds produced by microbes present in their habitat [27–31]. Sometimes these volatiles act additionally as oviposition cues that may provide the attracted female insects with information about the suitability of substrates for larval development [32].

The results from our study add to this evidence, and show that the growing mycelia of some species of fungi found in mushroom compost are attractive to gravid female *L*. *ingenua* and induce ovipostion. However, mycelia of other fungal species were not attractive and did not induce much oviposition, even in no-choice conditions. The goal of this research was to try to identify species that might elicit higher levels of attraction and oviposition from gravid female *L*. *ingenua* than to *T*. *aggressivum*, the pestiferous green mold fungus species already shown to be attractive to these females [22]. Among the species we tested, we found none that were more attractive than *T*. *aggressivum*. This fungal species thus must still be considered to have the most behaviorally active set of volatiles related to *L*. *ingenua* attraction and reproduction.

The growing mycelia from only a few species of fungi were as attractive as *T*. *aggressivum* in our study. These species were *S*. *thermophilium*, from the compost, and the *P*. *citrinum* that we found on adult female bodies. It is interesting that of these two species, only *P*. *citrinum* induced levels of oviposition equivalent to levels induced by *T*. *aggressivum* in addition to being as attractive to females as *T*. *aggressivum*. On the other hand, despite its attractiveness, *S*. *thermophilium* mycelia evoked very little oviposition from *L*. *ingenua* females even at seven days old in the no-choice arena. These results indicate distinct differences between this species’ volatile cues evoking significant levels of female *L*. *ingenua* attraction and the lack of cues that would induce females to oviposit.

Several fungal species were significantly less attractive to females than *T*. *aggressivum*, including *A*. *niger* that was phoretic on males, *A*. *flavus* that we isolated from larval frass, *A*. *fumigatus* found to be phoretic on adult females, and *Chaetomium* sp. Again in these cases there were some differences between attraction and the propensity to oviposit, but these differences were in the opposite direction from those in the *P*. *citrinum* and *S*. *thermophilium* results discussed above. Although females were less attracted to *A*. *flavus* compared to *T*. *aggressivum*, they were stimulated to oviposit more eggs on *A*. *flavus* than on *T*. *aggressivum* in two-choice oviposition tests.

These results might indicate that the volatiles from a suite of different fungal species act in concert in the natural setting of mushroom compost. Volatiles from some species such as *S*. *thermophilium* might cause gravid females to be attracted to the compost, and then volatiles from others, such as from *A*. *fumigatus* or *A*. *flavus*, might induce oviposition once females arrived at this area of compost, even though the volatiles from such species are not themselves very attractive to females. *Penicillium citrinum* seems to be similar in behavioral activity to *T*. *aggressivum* in being attractive to females as well as inducing high levels of ovipostion. It would be interesting to find out whether *T*. *aggressivum* is truly vectored by *L*. *ingenua* adults as has been conjectured. Then we might be able to see whether there is a relationship between fungal species known to be phoretic on *L*. *ingenua* adults—such as *P*. *citrinum* and possibly *T*. *aggressivum*—and high levels of both *L*. *ingenua* attraction and oviposition.

There is evidence of strong relationships in other dipteran-plant-host systems between microbial infection and reproductive behavior of adult females. Flies in the genus *Bradysia* (Diptera: Sciaridae), the darkwing fungus gnats, can be severe pests of ornamental and vegetable greenhouse production systems [33, 34]. Female *Bradysia impatiens* are more attracted to geranium seedlings infected with several plant-pathogenic *Pythium* spp. compared to healthy seedlings [35]. In the case of the onion maggot fly, *Delia antiqua* (Diptera: Anthomyiidae), adult females are more attracted to diseased compared to healthy onion plants and their larvae develop better on these damaged and diseased plants than on healthy ones [36]. Furthermore, insects from other orders vector specific fungal and other microbial symbionts to host substrates that assist the insect with nutrition acquisition and subsequently enhance its survival [37–39].

Field evaluations of dipteran adults attracted to traps baited with *Penicillium expansum* showed high catches of flies in the families Chironomidae, Drosophilidae, Sarcophagidae, and Syrphidae [40]. However none of the flies captured in these traps were from the fungus gnat families, e.g., Sciaridae, Mycetophilidae, or Phoridae. Some studies have shown that there may be microbial semiochemicals that attract larval dipterans. *Drosophila melanogaster* larvae have been shown to be attracted toward pure cultures of *P*. *expansum* under laboratory conditions [41].

A previous study found that *L*. *ingenua* will oviposit on *Aspergillus versicolor*, but that the hatched larvae do not survive past the first instar [42]. Similar results for *Aspergillus* species were seen in our choice and no-choice experiments for *A*. *flavus* and *A*. *fumigatus*, in which females would choose to lay eggs on the fungi but subsequent larval survival was zero. Larval survival on *P*. *citrinum* was fairly high (60%), corroborating previous results that *L*. *ingenua* can complete its life cycle on some *Penicillium* species [42]. It is interesting that both *P*. *citrinum* and *S*. *thermophilum* were equally as attractive to *L*. *ingenua* females as *T*. *aggressivum*, but that larvae could only complete their life cycle to adulthood on *P*. *citrinum* and *S*. *thermophilum*, not on *T*. *aggressivum*.

One explanation for *L*. *ingenua* attraction to the isolated fungal species, other than direct acquisition of nutrients, may be that the fungus provides some type of detoxification benefit to feeding larvae. Previous research suggests that fungal species in the genus *Attamyces*, cultivated by some species of ants, and *Symbiotaphrina* vectored by some cigarette beetle species, may provide some detoxifying qualities to their host insect [43]. The attractive fungal species in our study may also provide increased substrate utilization for *L*. *ingenua* larvae. It has been suggested that the Asian Longhorn beetle, *Anoplophora glabripennis*, contains a soft-rot fungal gut symbiont in the species complex *Fusarium solani/Nectria haematococca* that aids in the breakdown and metabolism of lignin [44]. Future work should examine whether any of the isolated species of fungi contain genes encoding for xylanases, proteinases, or cellulases, similar to such enzymes previously described for fungi associated with leaf cutting ants [45]. Enzymatic activity may provide information for the possible benefit to attracted and ovipositing female *L*. *ingenua* flies.

Several different fly species have been shown to carry *A*. *flavus*, *A*. *fumigatus*, *A*. *niger*, and *Penicillium* sp. spores on their bodies [46–50]. *L*. *ingenua* flies may be carrying spores of these fungi into mushroom houses from outside and act as a source of external inoculum. They may also act as vectors that spread the spores of these fungi between different mushroom growing rooms in a mushroom house. More efforts should be made to investigate a possible link between *L*. *ingenua* flies and these human pathogenic and allergenic fungi in mushroom production systems [51–57].

This research offers insight into potential sources of fungi in mushroom compost that elicit attraction and oviposition to the damaging sciarid fungus gnat, *L*. *ingenua*. In the future we hope to identify attractive head-space volatiles for the species of fungi we isolated, focusing specifically on those that were most attractive to female flies, i.e., *T*. *aggressivum*, *P*. *citrinum* isolated from adult female flies, and *S*. *thermophilium* found in compost. By isolating and identifying any volatiles emitted by these attractive species of fungi, it may be possible to develop some kind of synthetic lure that could be used in a mushroom growing house for monitoring and control of host-seeking female flies. *Lycoriella ingenua* are known to be attracted to mushroom compost the moment a mushroom house is filled with spawned compost, and the number of invading female flies decreases after 14 days [58]. Thus, during the period in which a mushroom crop develops in the compost, *L*. *ingenua* survival significantly decreases [9, 10, 59]. If we can prevent this initial invading population within the first 14 days via some kind of attract and kill strategy, all other invading populations will not likely establish in the compost and cause damage to the mushroom crop.
